# A Randomized, Double‐Blind, Placebo‐Controlled Trial Comparing the Effects of Commercially Available Ubiquinone, Ubiquinol, and Geranylgeraniol Dietary Supplements on Plasma CoQ10 Concentrations

**DOI:** 10.1155/jnme/5589963

**Published:** 2026-09-27

**Authors:** Christopher R. D’Adamo, Michelle Leary, Christopher Mote, Ronald Ackerman, Mark Holthouse, Brandilyn Binstock, Ellie Adair, Gabriela Piedrahita, Scott Wehage, Kristine Burke

**Affiliations:** ^1^ Department of Family and Community Medicine, University of Maryland School of Medicine, Baltimore Maryland, 21201, USA, umaryland.edu; ^2^ OvationLab, Richmond Virginia, 23220, USA; ^3^ Vida Integrated Health, Seattle Washington, 98122, USA; ^4^ Cornerstone Health Community, Centennial Colorado, 80111, USA; ^5^ Zenacare Research, Miami Florida, 33169, USA; ^6^ Functional Medicine of Idaho, Meridian Idaho, 83642, USA; ^7^ True Health Center for Precision Medicine, El Dorado Hills California, 95762, USA

**Keywords:** coenzyme Q10, geranylgeraniol, ubiquinol, ubiquinone

## Abstract

**Background:**

Coenzyme Q10 (CoQ10) plays a fundamental role in energy production and antioxidant activity in humans. Direct supplementation with both the ubiquinol and ubiquinone forms of CoQ10 increases circulating concentrations, although comparative efficacy remains uncertain. Furthermore, nutrients supporting endogenous CoQ10 production, such as geranylgeraniol (GG), suggest that supplementation may indirectly increase CoQ10 concentrations.

**Methods:**

A randomized, double‐blind, parallel‐arm, placebo‐controlled trial was conducted comparing 8 weeks of label‐dose supplementation with commercially available formulations of (1) ubiquinone; (2) ubiquinol; (3) GG; (4) ubiquinol + GG; and (5) placebo among healthy adults. The primary outcome was plasma CoQ10 concentrations. Secondary outcomes included lipid concentrations, markers of kidney and liver function, and validated questionnaires (PROMIS–Global Health, Profile of Mood States–Vigor subscale, and SF‐36 Vitality subscale) assessing physical and mental health. Mean changes were compared across study arms using analysis of covariance (ANCOVA). Bonferroni correction for multiple comparisons was applied to the comparisons to placebo, and statistical significance was set at *p* < 0.0125. Tukey’s multiple comparison procedure was applied to comparisons between each of the active study products, and statistical significance was set at *p* < 0.05.

**Results:**

A total of 150 participants were randomized. The mean age was 52.1 years, and the sample was diverse with respect to sex and race/ethnicity. Changes in CoQ10 with ubiquinone (+ 1.46), ubiquinol (+ 2.29), and ubiquinol + GG (+ 2.45) were greater than placebo (*p* < 0.0001). There were no differences between GG and placebo, ubiquinone and ubiquinol, or ubiquinol and ubiquinol + GG (*p* ≥ 0.15). Ubiquinol + GG (+ 0.99 mg/L) outperformed ubiquinone (*p* = 0.045). There were no differences versus placebo in questionnaires (*p* > 0.16). Adverse events were rare across all study arms.

**Conclusions:**

Supplementation with ubiquinone, ubiquinol, and ubiquinol + GG was well‐tolerated and increased CoQ10 concentrations among healthy adults. While GG had no independent effects on CoQ10 concentrations, an interaction effect cannot be excluded, and this should be evaluated in a future factorial trial specifically designed for this purpose. Future studies evaluating the impact of ubiquinone + GG may also be warranted.

**Trail Registration:** ClinicalTrials.gov: NCT06640465

## 1. Introduction

Coenzyme Q10 (CoQ10) is a naturally occurring compound that serves as an essential component in cellular energy production and antioxidant defense systems throughout the human body [1]. CoQ10 is primarily located in the inner mitochondrial membrane, where it plays an indispensable role as an electron carrier in the mitochondrial electron transport chain, shuttling electrons from Complexes I and II to Complex III, thereby facilitating ATP synthesis. This integral function in bioenergetics is further complemented by CoQ10’s potent antioxidant properties. CoQ10 cycles between its oxidized (ubiquinone) and reduced (ubiquinol) forms to scavenge free radicals and protect cellular membranes from oxidative damage [2]. The molecular structure of CoQ10 affords it the ability to integrate into lipid bilayers and interact with various cellular enzymes, ensuring efficient energy production and stabilization of membranes.

The critical role of CoQ10 is underscored by an extensive body of evidence that has demonstrated that lower levels of CoQ10 are associated with impaired mitochondrial function [3], increased oxidative stress [4], and a host of age‐related disorders, including cardiovascular disease, neurodegenerative conditions, dermatological manifestations of aging, and others [5]. Supplementation with CoQ10 has also demonstrated clinical benefits. Studies in chronic heart failure and coronary artery disease populations have reported reductions in inflammatory biomarkers [6], hospitalization rates [7], and mortality following CoQ10 supplementation [8]. Beyond the cardiovascular realm, clinical studies have also reported beneficial effects in other domains such as female [9] and male fertility [10], skin health [11], migraine prevention [12], and metabolic health [13, 14].

Despite the unequivocal importance of CoQ10 in cellular physiology and clinical evidence, considerable uncertainty remains regarding the most efficacious interventions to increase CoQ10 levels in the body. CoQ10 is predominantly commercially available in the two interconvertible forms—the oxidized ubiquinone and the reduced ubiquinol—each of which exhibits distinct biochemical characteristics that influence absorption, bioavailability, and subsequent clinical efficacy. Ubiquinone, which has been the traditional choice for supplementation due to its chemical stability and extensive documentation in early clinical studies, requires in vivo enzymatic reduction to become active [2], a conversion process that might be less effective in individuals with compromised mitochondrial function. In contrast, ubiquinol is already in its reduced state [2], theoretically allowing for more rapid absorption and immediate antioxidant activity, which could be especially beneficial in mitigating oxidative stress in conditions like heart failure and endothelial dysfunction. However, while several review articles have suggested that the enhanced bioavailability of ubiquinol may confer superior pharmacokinetic profiles—resulting in higher plasma CoQ10 concentrations [1, 15]—these purported advantages have been met with mixed results in clinical studies. Despite ubiquinol’s increased plasma levels in older populations, a recent systematic review and meta‐analysis of 33 trials revealed that clinical outcomes such as improvements in cardiac function, reduction in hospitalization rates, and overall mortality benefits appear comparably favorable when supplementing with ubiquinone [7], which benefits from a broader evidence base and more extensive clinical evaluation. While ubiquinol may offer advantages in terms of pharmacokinetics, the heterogeneity of study designs, dosing regimens, and patient populations precludes a definitive conclusion regarding clinical superiority over ubiquinone. Moreover, the interconversion between the two forms in vivo, influenced by the activity of oxidoreductases and the need for ubiquinol to first undergo oxidation to ubiquinone in the gastrointestinal tract prior to re‐reduction before entering circulation [1, 2], adds another layer of complexity to this debate.

In parallel with direct CoQ10 supplementation strategies, recent advances have spotlighted the role of natural products in enhancing endogenous CoQ10 biosynthesis. Geranylgeraniol (GG) has emerged as a particularly promising agent due to its function as an isoprenoid precursor in the mevalonate pathway, a crucial metabolic cascade responsible for the synthesis of CoQ10 [16, 17]. Naturally abundant in select plant sources such as annatto, GG contributes structurally to the polyisoprenoid chain of CoQ10 and thereby facilitates its proper integration into the mitochondrial membrane. Supplementation with GG has been shown to restore mevalonate‐pathway intermediates and regenerate CoQ10 synthesis, leading to increased cellular CoQ10 levels and improved mitochondrial function [16, 18]. GG may not only replenish CoQ10 stores but also provide additional cytoprotective benefits by exerting anti‐inflammatory [19, 20], antiatrophy [21], and antiapoptotic effects [22]. Furthermore, GG has been shown to positively impact mitochondrial function (increased mitochondrial respiration, mitogenesis, and mitophagy) [23].

Despite the considerable body of evidence supporting both direct supplementation with CoQ10 and the potential of natural products such as GG to indirectly enhance endogenous CoQ10 synthesis, no randomized, head‐to‐head, placebo‐controlled trials have compared commercially available supplements with direct and/or indirect CoQ10‐enhancing properties. This deficiency in comparative evidence currently limits the ability for clinicians and patients to discern which supplementation strategy—whether it be direct administration of ubiquinone, ubiquinol, the use of endogenous production supporting products such as GG, or a combined approach of direct and indirect support—yields the most robust improvements in circulating CoQ10 concentrations. Recognizing this unmet need, a five‐arm, randomized, double‐blind, placebo‐controlled clinical trial comparing supplementation with common, commercially available formulations of ubiquinone, ubiquinol, GG, and the combination of ubiquinol and GG on plasma CoQ10 levels and a variety of metabolic, lipid, and patient‐reported quality of life outcomes was conducted. The research team hypothesized that while all of the natural products would increase plasma CoQ10 concentrations versus placebo, the combination of ubiquinol and GG would have the greatest increases.

## 2. Materials and Methods

### 2.1. Study Design

An 8‐week, randomized, double‐blind, parallel‐arm, placebo‐controlled clinical trial was conducted comparing the effects of commercially available (1) ubiquinone; (2) ubiquinol; (3) GG; (4) combination ubiquinol plus GG dietary supplements; and (5) placebo on CoQ10 concentrations and validated questionnaires of physical health, mental health, energy, and fatigue.

The clinical trial was approved by Pearl IRB (Approval #: 2024‐0382) and was prospectively registered at ClinicalTrials.gov (NCT06640465) with submission on October 10, 2024, first participant enrolled on October 12, 2024, and public posting on October 15, 2024. The interval between submission and public posting reflects the National Library of Medicine’s standard quality‐control review of registry records. The study was conducted in alignment with the principles outlined in the 2024 Declaration of Helsinki. The manuscript was composed in accordance with the CONSORT statement. The deidentified, raw study data are publicly available at Figshare (https://doi.org/10.6084/m9.figshare.31386814).

### 2.2. Setting and Participants

Participants were recruited from several clinical sites across the United States from October 2024 through June 2025. Interested participants were screened by staff at the clinical sites for eligibility on the inclusion/exclusion criteria described below. Signed informed consent was obtained from all participants electronically.

#### 2.2.1. Inclusion Criteria

- Adult females or males age ≥ 40 years
- Ability to read and speak English

#### 2.2.2. Exclusion Criteria

- Daily use in the past 2 months of any dietary supplements containing CoQ10 or GG
- Taking a statin medication in the past 6 months
- Current daily usage of H2 blockers or PPI medications
- Current daily use of bisphosphonates
- Current daily tobacco smoker
- Known allergies to any substance in the study products
- Currently pregnant, lactating, or planning to become pregnant in the next 12 weeks
- Any medical condition deemed to be contraindicated for the study products

### 2.3. Randomization and Allocation Concealment

The random allocation sequence was generated by the study statistician using SAS Version 9.4 (SAS Institute Inc., Cary, NC) with a computerized random number generator and permuted blocks of size five in a 1:1:1:1:1: allocation.

Allocation concealment was maintained through centralized product labeling and distribution. The SAS‐generated randomization schedule for the five study arms was provided only to the product distribution center that labeled and shipped the corresponding study products using participant IDs. The clinical sites and study investigators that enrolled participants had no access to the randomization list or study arm allocation.

### 2.4. Blinding

All study products were dispensed in identically appearing bottles labeled only with participant ID and study information. The individual capsules from each commercial product were not modified or over‐encapsulated. The randomization list was accessible only to the central product distribution center, while clinical site investigators, study staff involved in participant interaction, laboratory personnel, and statistical analysts remained blinded to treatment allocation until the database was locked and all of the statistical analyses were completed.

### 2.5. Interventions

Enrolled participants were randomized to placebo or one of the following commercially available dietary supplements: ubiquinone (CoQ10 200 mg, NOW Foods, Bloomingdale, IL, USA), ubiquinol (QH‐Absorb 200 mg, Jarrow Formulas, Sherman Oaks, CA, USA), GG (Annatto‐GG 300 mg, Designs for Health, Palm Coast, FL, USA), or ubiquinol and GG combination (CoQNol 200 [200 mg ubiquinol and 120 mg GG], Designs for Health, Palm Coast, FL, USA). The product labels are provided in the Supporting Information (Figure SF1).

At baseline, the participant had a blood draw at a clinical site and completed several validated quality of life questionnaires. The participant was then drop‐shipped their study product from the centralized product distribution center.

The intervention consisted of taking one capsule per day of the commercially available product (the label dosage for all active products) or placebo with a meal for 8 weeks. The label dose was administered to best support the safety of study participants, since each of the products in the study has been commercially available with no serious adverse events reported to the United States Food and Drug Administration. The research team believed that the label dose would provide the most relevant and clinically actionable comparison for researchers, clinicians, and patients. Participants were explicitly instructed to take the product with a meal due to the lipid solubility of CoQ10 and GG.

Participants were assigned a research associate, who they were instructed to contact if they had any questions related to the study or adverse events to report. The research associate also reached out to the study participants as they approached the conclusion of the study.

After taking the study product for 8 weeks, the participant returned to the clinical site for their end‐of‐study blood draw and completed the validated questionnaires. Adherence to the assigned study product was also assessed by self‐report at the end of the study. Participants were asked to estimate the proportion of doses taken over the 8‐week study period, and adherence was summarized as the percentage of doses reported to have been consumed. An adherence threshold of 80% of prescribed doses taken was prespecified to define acceptable adherence for descriptive and potential per‐protocol sensitivity analysis considerations.

### 2.6. Outcomes

#### 2.6.1. Laboratory Outcomes

Fasted blood draws were collected from participants at the clinical sites at baseline and at the conclusion of the 8‐week study.

Primary outcome: Plasma CoQ10 concentrations, measured by ultra‐performance liquid chromatography with ultraviolet detection (UPLC/UV).

Secondary outcomes: (1) Lipids and apolipoproteins panel—total cholesterol (TC), direct low‐density lipoprotein cholesterol (LDL‐C), high‐density lipoprotein cholesterol (HDL‐C), triglycerides (TG), nonhigh‐density lipoprotein cholesterol (non‐HDL‐C), very‐low‐density lipoprotein cholesterol (VLDL‐C), and lipid ratios (total cholesterol/high‐density lipoprotein cholesterol [TC/HDL‐C], very‐low‐density lipoprotein cholesterol/triglycerides [VLDL‐C/TG], high‐density lipoprotein cholesterol/triglycerides [HDL‐C/TG]); (2) Chemistry Panel—fasting glucose, blood urea nitrogen (BUN), creatinine, electrolytes (sodium, potassium, chloride, carbon dioxide [CO_2_], and anion gap), total protein, albumin, calcium, total bilirubin, aspartate aminotransferase (AST), alanine aminotransferase (ALT), and alkaline phosphatase; and (3) Kidney indices—blood urea nitrogen/creatinine (BUN/creatinine) ratio, estimated glomerular filtration rate (eGFR).

All analyses were performed at Boston Heart Diagnostics (Framingham, MA, USA), a CLIA‐certified laboratory.

#### 2.6.2. Questionnaire Outcomes

The following validated questionnaires were secondary outcomes assessed at baseline and at the conclusion of the 8‐week study.

Patient‐Reported Outcomes Measurement Information System (PROMIS)–Global Health: The PROMIS Global Health is a 10‐item, patient‐reported questionnaire that assesses general health‐related quality of life compared with normal values for the general population [24]. These questions are designed to broadly assess aspects of health‐related quality of life, including physical functioning, pain, fatigue, emotional distress, social functioning, cognitive functioning, mental health, role functioning, and overall perceived health. Responses are given on 5‐point Likert‐type scales (e.g., from *poor* to *excellent* or from *never* to *always*), and items are scored and summed according to the PROMIS Global Health scoring manual to generate two summary scores: a Global Physical Health raw score and a Global Mental Health raw score. These raw scores are then converted to standardized *T* scores (mean = 50, standard deviation = 10) based on PROMIS general population norms, such that a *T* score of 50 represents the average level of health in the reference population and each 10‐point difference represents one standard deviation. Higher *T* scores on the Global Physical Health and Global Mental Health scales indicate better health‐related quality of life.

Profile of Mood States (POMS)—Vigor Subscale: The POMS is a widely used questionnaire that assesses an individual’s mood states. The Vigor subscale is one of the six subscales that measure the intensity of various mood states [25, 26]. The Vigor subscale questions are designed to assess the degree to which an individual feels energetic, lively, and full of energy. These questions are rated on a 5‐point Likert scale, ranging from 0 (*not at all*) to 4 (*extremely*). The scores on these questions are then used to calculate the Vigor subscale score, which can range from 0 to 32. A higher score indicates a more positive mood state, characterized by feelings of energy and vitality.

Short Form Health Survey (SF‐36)–Vitality Subscale: The SF‐36 is a widely used set of health‐related quality of life measures. It includes eight subscales, one of which is the Vitality subscale [27, 28]. The Vitality subscale of the SF‐36 measures energy and fatigue. It consists of four questions that assess how an individual feels about their energy levels and fatigue over the past 4 weeks. Responses are rated on Likert‐type scales (ranging from *all of the time* to *none of the time*), recoded according to the SF‐36 scoring manual, summed, and linearly transformed to a 0–100 scale. Higher scores indicate greater vitality and lower fatigue.

### 2.7. Sample Size Calculation

Assuming *α* = 0.0125 (this threshold accounts for Bonferroni correction for multiple comparisons between the four active products and placebo under study, as described in the Statistical Analysis section below), 80% power, a standard deviation in the difference from baseline to end of study of 1 mg/L, and a minimal detectable difference of 1 mg/L between study arms, a total sample size of 120 participants to complete the trial was needed (24 participants in each arm).

In order to account for a conservative and higher than expected dropout rate of over 20%, 150 participants (30 participants per arm) were enrolled to achieve the final target sample size.

### 2.8. Statistical Analysis

Descriptive statistics were computed to provide baseline characteristics of the sample by study arm. Normality of continuous variables was assessed using Kolmogorov–Smirnov tests. Baseline characteristics were compared across the five study arms utilizing ANOVA (normally distributed variables), the Kruskal–Wallis test (non‐normally distributed variables), and chi‐square tests (categorical variables).

The primary and secondary analyses were conducted using a complete‐case approach. For each outcome, all randomized participants with both baseline and end‐of‐study measurements for that outcome were included. All participants were analyzed according to their randomized assignment, and no postrandomization exclusions related to adherence or any other factors were applied. Mean changes in the primary and secondary outcomes from baseline to the end of the study were compared across study arms using analysis of covariance (ANCOVA), which accounts for baseline values of the outcome being analyzed. Tukey’s post hoc pairwise tests were also utilized to compare outcomes between each of the five study arms. No imputation was performed for participants missing end‐of‐study data.

Considering the four active arms being compared to placebo in the primary study analysis, a Bonferroni correction for multiple comparisons was applied, and statistical significance was set at *p* < 0.0125. Statistical significance for the comparisons between each of the active study products with Tukey’s pairwise tests, which account for family‐wise error rate across all pairwise comparisons and thus do not require further Bonferroni correction, was set at *p* < 0.05. All statistical analyses were performed in SAS Version 9.4 (SAS Institute Inc., Cary, NC, USA).

## 3. Results

150 participants enrolled and were randomized to one of the five study arms. The participant flow from screening to inclusion in the primary analysis is shown in the CONSORT diagram in Figure 1, with reasons for exclusion provided at each stage.

**FIGURE 1:**
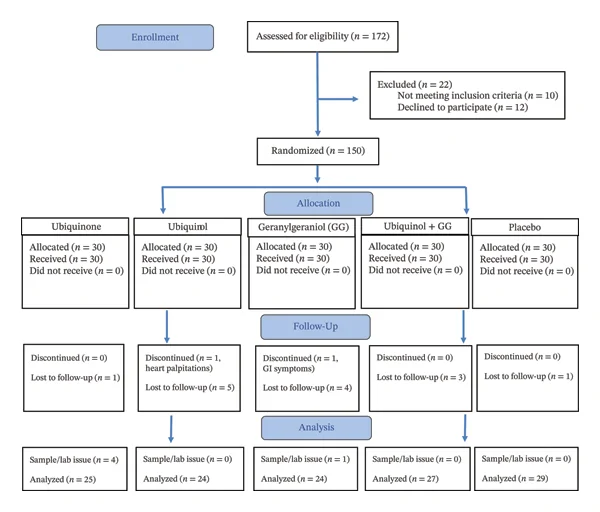
CONSORT 2025 flow diagram.

The characteristics of the study population by study arm are provided in Table 1. The mean (standard deviation) age of the study sample was 52.1 (8.4) years, and there were more females (74.7%) than males (25.3%). The study sample was diverse with respect to race/ethnicity, with 56.7% White/not Hispanic or Latino, 31.3% White/Hispanic or Latino, 7.3% Black/African American, 3.3% Asian, and 1.4% other. There were no differences between study arms in any demographic characteristics (*p* > 0.11).

**TABLE 1:** Characteristics of study population at baseline.

	Ubiquinone	Ubiquinol	Geranylgeraniol (GG)	Ubiquinol + GG	Placebo	*p* value^∗^
Demographics						
Age	52.4 (8.5)	52.3 (7.8)	49.1 (6.1)	51.6 (7.7)	55.0 (10.7)	0.11
Sex	Female = 20 (66.7%)	Female = 23 (76.7%)	Female = 26 (86.7%)	Female = 20 (66.7%)	Female = 23 (76.7%)	0.35
	Male = 10 (33.3%)	Male = 7 (23.3%)	Male = 4 (13.3%)	Male = 10 (33.3%)	Male = 7 (23.3%)	
Race	White/not Hispanic = 15 (50.0%)	White/not Hispanic = 18 (60.0%)	White/not Hispanic = 17 (56.7%)	White/not Hispanic = 18 (60.0%)	White/not Hispanic = 17 (56.7%)	0.61
	White/Hispanic = 10 (33.3%)	White/Hispanic = 8 (26.7%)	White/Hispanic = 10 (33.3%)	White/not Hispanic = 18 (60.0%)	White/Hispanic = 9 (30.0%)	
	Black/African American = 5 (16.7%)	Black/African American = 1 (3.3%)	Black/African American = 1 (3.3%)	Black/African American = 2 (6.7%)	Black/African American = 2 (6.7%)	
	Asian or Other = 0 (0.0%)	Asian or Other = 3 (10.0%)	Asian or Other = 2 (6.6%)	Asian or Other = 0 (0.0%)	Asian or Other = 2 (6.7%)	
Lipids						
Total cholesterol	204.1 (38.1)	205.4 (28.0)	207.5 (43.5)	213.0 (48.5)	207.7 (33.6)	0.93
Low‐density lipoprotein (LDL)	134.0 (32.4)	127.6 (25.9)	132.2 (39.0)	134.9 (45.5)	127.3 (24.0)	0.87
Very low‐density lipoprotein (VLDL)	15.9 (9.1)	14.4 (8.4)	16.1 (9.7)	20.8 (22.4)	11.7 (7.2)	0.11
High‐density lipoprotein (HDL)	55.6 (14.4)	63.3 (20.6)	60.0 (14.8)	58.2 (15.6)	65.9 (15.6)	0.14
Triglycerides	106.3 (61.4)	99.8 (48.7)	101.6 (59.1)	108.7 (75.7)	83.0 (32.4)	0.47
Liver function						
Alanine aminotransferase (ALT)	25.9 (19.4)	22.0 (14.7)	19.6 (6.6)	24.1 (12.1)	19.9 (9.0)	0.30
Aspartate aminotransferase (AST)	22.2 (7.1)	21.5 (6.7)	20.0 (5.5)	22.5 (6.5)	21.8 (6.5)	0.64
Alkaline phosphatase (ALP)	76.3 (17.0)	71.1 (20.9)	74.5 (18.2)	68.6 (15.4)	74.0 (24.8)	0.60
Total bilirubin	0.57 (0.26)	0.56 (0.27)	0.55 (0.47)	0.54 (0.28)	0.65 (0.33)	0.74
Kidney function						
Estimated glomerular filtration rate (eGFR)	90.0 (13.5)	91.8 (14.4)	91.4 (17.7)	91.3 (12.2)	86.8 (16.4)	0.70
Blood urea nitrogen (BUN)	14.1 (3.6)	14.5 (4.0)	14.6 (3.6)	15.2 (4.5)	14.9 (4.3)	0.86
Creatinine	0.86 (0.13)	0.83 (0.17)	0.84 (0.21)	0.86 (0.16)	0.86 (0.18)	0.87

^∗^ *p* values calculated by analysis of variance (ANOVA) for continuous variables and chi‐square tests for categorical variables. Statistical significance was defined as *p* < 0.05.

There were also no differences between the study arms in mean concentrations of lipids, markers of liver function, or markers of kidney function (*p* > 0.11) at baseline.

Adverse events were rare across all study arms, with only three adverse events reported in the study: *n* = 1 ubiquinone arm (gastrointestinal distress), *n* = 1 ubiquinol arm (heart palpitations), and *n* = 1 GG arm (gastrointestinal distress). Two participants dropped out due to adverse events (*n* = 1 GG arm [gastrointestinal symptoms] and *n* = 1 in the ubiquinol arm [heart palpitations]). There were no serious adverse events requiring medical treatment in the study. All study participants reported meeting the ≥ 80% adherence threshold in taking their assigned study product as defined in the protocol.

### 3.1. Primary Analysis—CoQ10 Concentration Comparisons vs. Placebo

Table 2 provides the plasma CoQ10 concentrations at baseline and the end of the study. There were no differences in CoQ10 concentrations between study arms at baseline (ANOVA F statistic = 0.041, *p* = 1.0). In the primary ANCOVA analyses for change in plasma CoQ10, accounting for baseline CoQ10 concentrations, the overall effect of study arm was significant (ANCOVA F statistic = 22.28, *p* < 0.0001). The changes in CoQ10 concentration with ubiquinone (+ 1.46), ubiquinol (+ 2.29), and ubiquinol + GG (+ 2.45) supplementation were all greater than placebo (*p* < 0.0001). There was no difference in the change in CoQ10 concentrations between the GG and placebo arms (*p* = 0.98).

**TABLE 2:** Mean CoQ10 concentrations (in mg/L) at baseline and end of study.

Study arm	Baseline	End of study	Baseline to end‐of‐study change	*p* vs. placebo^∗^
Placebo	0.93 (0.34)	0.96 (0.32)	+ 0.03 (0.47)	—
Ubiquinone	0.95 (0.27)	2.41 (1.31)	+ 1.46 (1.03)	< 0.001
Ubiquinol	0.94 (0.30)	3.24 (1.26)	+ 2.29 (1.06)	< 0.001
GG	0.96 (0.30)	0.95 (0.33)	−0.01 (0.46)	0.98
Ubiquinol + GG	0.95 (0.33)	3.40 (1.21)	+ 2.45 (1.07)	< 0.001

^∗^ *p* values calculated by analysis of covariance (ANCOVA). Statistical significance was defined as *p* < 0.0125 to reflect four comparisons to placebo in the Bonferroni adjustment.

### 3.2. Secondary Analysis–CoQ10 Concentration Comparisons Between Commercially Available Products

The Tukey pairwise comparisons between active study arms are provided in Table 3. In brief, the ubiquinone (+ 1.47 mg/L), ubiquinol (+ 2.30 mg/L), and ubiquinol + GG (+ 2.46 mg/L) arms all outperformed the GG arm (*p* < 0.0001). There was no difference between the ubiquinone and ubiquinol arms (*p* = 0.15) or the ubiquinol and ubiquinol + GG arms (*p* = 0.99). However, the ubiquinol + GG arm (+ 0.99 mg/L) outperformed the ubiquinone arm (*p* = 0.045).

**TABLE 3:** Comparisons of mean CoQ10 changes between active study arms.

Comparison	Mean difference	95% CI	*p* value^∗^
Ubiquinone vs. GG	1.47	0.46–2.49	< 0.0001
Ubiquinol vs. GG	2.30	1.28–3.32	< 0.0001
Ubiquinol + GG vs. GG	2.46	1.46–3.46	< 0.0001
Ubiquinol + GG vs. ubiquinol	0.16	−0.81–1.13	0.99
Ubiquinol vs. ubiquinone	0.83	−0.17–1.82	0.15
Ubiquinol + GG vs. ubiquinone	0.99	0.014–1.96	0.045

^∗^ *p* values calculated by Tukey’s pairwise *t*‐tests, which account for multiplicity of pairwise comparisons. Statistical significance was defined as *p* < 0.05.

### 3.3. Secondary Analysis—Other Biomarkers

There were no differences in the changes in mean lipid concentrations or markers of liver function between any active intervention arm and placebo from baseline to the end of the study in the ANCOVA analyses (*p* > 0.45). There were unadjusted, hypothesis‐generating signals of differences in changes in markers of kidney function between study arms after accounting for baseline levels, which favored the GG and ubiquinol + GG arms. The GG arm had an improvement in BUN versus placebo (−1.59, 95% CI [−2.84, −0.35], *p* = 0.0008) and the ubiquinol + GG arm had improvements in eGFR (+ 4.70 mL/min/1.73 m^2^, 95% CI [1.45, 7.94], *p* = 0.0099) and creatinine (−0.043 mg/dL, 95% CI [−0.078, −0.0075], *p* = 0.049) versus placebo from baseline to the end of the study.

### 3.4. Secondary Analysis—Questionnaire Outcomes

ANCOVA analyses revealed no significant differences between any active intervention arm and placebo with respect to changes in the mean scores of the validated patient‐reported outcome measures (PROMIS–Global Health, POMS–Vigor, SF‐36–Vitality) from baseline to the end of the study (*p* > 0.44) (Supporting Information, Table S1).

## 4. Discussion

This clinical trial provided further evidence that supplementation with commercially available forms of either the oxidized (ubiquinone) or reduced (ubiquinol) forms of CoQ10 effectively increases plasma CoQ10 concentrations among healthy adults across a broad age range. While GG supplementation alone did not have an impact on CoQ10 concentrations and the study was not able to assess an interaction effect, an interaction effect cannot be excluded in supplementation with ubiquional and GG, and this should be evaluated in a future factorial trial specifically designed for this purpose. In addition, the unadjusted, hypothesis‐generating signal of ubiquinol and GG positively impacting kidney function may warrant additional studies. All products studied were generally well tolerated over the 8‐week study period, as reflected by the combination of a lack of deleterious changes in liver or kidney function, very few adverse events, and no serious adverse events in any study arm.

The lack of any meaningful difference in the change in CoQ10 concentrations between supplementation with ubiquinone or ubiquinol noted in this study is supported by both mechanistic and clinical trial data. Mechanistically, both mandatory lymphatic redox conversion and the inherent oxidative instability of ubiquinol in the gastrointestinal tract may explain these findings. Following oral ingestion, ubiquinol undergoes oxidation to ubiquinone in the stomach and duodenum and then requires subsequent re‐reduction in mesenteric lymphatics before entering circulation [1]. Conversely, ubiquinone remains stable during gastric transit and undergoes enzymatic reduction to ubiquinol in the lymphatic system [29, 30]. Human pharmacokinetic studies demonstrate that regardless of the ingested form, CoQ10 appears in peripheral blood predominantly as ubiquinol (approximately 95% of total CoQ10), with no significant differences in redox status between formulations [31]. Clinical evidence is mixed, with some studies favoring ubiquinol [32, 33] and others favoring ubiquinone [31], and it has been argued that age may confound the effects. Although even among older adults specifically, some clinical studies have found a superior increase in CoQ10 concentrations with ubiquinol supplementation, and other comparative bioavailability studies reveal that ubiquinone significantly increased CoQ10 concentrations and ubiquinol did not [31]. Furthermore, crossover trials in healthy adults across the lifespan demonstrate that intestinal absorption variability is independent of CoQ10 redox form, with intersubject variation exceeding formulation differences [34]. This trial adds to the evidence base that commercially available formulations of ubiquinone and ubiquinol are both effective at increasing CoQ10 concentrations.

The absence of an independent effect of GG on circulating CoQ10 in this trial, despite a signal for superiority of the ubiquinol + GG arm versus ubiquinone, is biologically plausible when GG is viewed primarily as a conditional precursor and pathway modulator rather than a stand‐alone CoQ10 secretagogue. As an isoprenoid substrate in the mevalonate pathway, GG donates the geranylgeranyl side chain required for CoQ10 biosynthesis [35], but overall flux through this pathway is tightly regulated at HMG‐CoA reductase and other upstream points [36]. In a relatively healthy sample in the normal range of plasma CoQ10 concentrations, like in this study that is not on statin therapy, endogenous synthesis may not be substrate‐limited at the level of GG. Thus, supplementation with GG alone over 8 weeks may not be expected to produce large changes in plasma CoQ10. Moreover, plasma CoQ10 primarily reflects hepatic handling and lipoprotein transport of exogenous CoQ10, while modest GG‐driven increases in intracellular CoQ10 could remain compartmentalized within mitochondria and membranes and thus fall below the limit of detection in the plasma assays of this study.

As a rationale for future research and in contrast to independent administration of GG, when GG is co‐administered with an exogenous source of CoQ10 (ubiquinol, in this study), several mechanisms could support a synergistic effect relative to ubiquinone. Ubiquinol provides an immediate and substantial exogenous CoQ10 load into chylomicrons and lipoproteins, while GG may simultaneously expand the intracellular pool of prenylated CoQ10 by supplying additional geranylgeranyl side chains for de novo synthesis [18]. This combined direct/exogenous and indirect/endogenous support could modestly raise the steady‐state total CoQ10 pool above that achieved with exogenous CoQ10 alone, even if the GG contribution is insufficient when GG is given in isolation. In addition, GG participates in protein geranylgeranylation and may influence the function or membrane localization of enzymes and transporters involved in CoQ10 trafficking, redox cycling, and lipoprotein metabolism [22]. It is plausible that these effects only become functionally relevant when ubiquinol substrate is present in abundance, potentially enhancing incorporation or retention of CoQ10 in lipoprotein particles and peripheral tissues.

An analogous pattern has been documented with other natural products, in which one compound has little or no independent effect on a biomarker but markedly potentiates the effect of an accompanying compound. A well‐characterized example is the combination of curcumin and piperine. Piperine alone does not raise plasma curcumin concentrations, but co‐administration of curcumin with piperine greatly increases curcumin bioavailability [37]. Another mechanistically analogous example is the combination of sesame lignans with *γ*‐tocopherol, where lignans alone lack vitamin *E* activity but significantly potentiate *γ*‐tocopherol’s antioxidant effects by inhibiting its metabolism, leading to enhanced tissue levels and biomarker improvements [38].

In addition to the randomized, double‐blind, placebo‐controlled design and use of standard laboratory and validated questionnaire outcomes, a key strength of this trial was the use of commercially available dietary supplements used at label doses, which increases the real‐world applicability of the findings. Unlike research‐grade or custom‐formulated products that may not be accessible to consumers or clinicians, the formulations evaluated in this study were popular products that are currently available on the market. This enhances the external generalizability of the study, supporting the pragmatic relevance of the findings for clinicians and consumers when choosing between commonly marketed CoQ10 and GG formulations.

While the findings of this trial contribute to the literature on commercially available options purported to exogenously or endogenously support CoQ10 levels in healthy adults, there are several limitations of the study that are worthy of mentioning. First, the 8‐week study duration may not have been sufficient to discern differences in the patient‐reported outcomes. While 8 weeks is ample to detect differences in CoQ10 concentrations, more time with supplementation may be required to fully capture the downstream clinical effects of the products on functional outcomes such as fatigue, vigor, and overall physical and mental health. This may be particularly pertinent in the generally healthy, middle‐aged study population of this study. Another limitation is the formulation differences among the commercially available products. While the research question was specifically centered around commercially available products to enhance the external generalizability and real‐world relevance to patient care, CoQ10 bioavailability is influenced by other formulation factors, specifically crystal dispersion status and carrier lipid composition [39]. The vast majority of commercially available ubiquinone products are crystalline with no carrier lipids, including the best‐sellers on most outlets where dietary supplements are commonly purchased by practitioners and patients. Accordingly, a common crystalline ubiquinone product was chosen for the study, whereas the ubiquinol and ubiquinol + GG formulations were in lipid‐containing gelcaps. By design, all of these products included medium chain triglycerides to minimize any potential differences in bioavailability by different carrier lipids. While this may have had a potential influence favoring the ubiquinol formulations over the ubiquinone formulation, participants were explicitly instructed to consume their study product with a meal, which typically provides considerably more fat for the lipid‐soluble CoQ10 than the contents of the capsules in the ubiquinol and ubiquinol + GG arms of the study (less than 1 g of fat). In any case, the lack of difference in effect on CoQ10 concentrations between commercially available ubiquinone and ubiquinol formulations withstood this difference in lipid content in the capsules. Beyond lipid content, the bioavailability of CoQ10 may also be influenced by differences in delivery system and other formulation characteristics [40] that vary across finished products. Another limitation of the study is that it is also possible that unblinding at the participant level may have occurred because the products were not re‐encapsulated to make them identical across study arms. While the bottles provided to participants were identical across study arms, the study capsules dispensed to participants were not altered, over‐encapsulated, or otherwise modified in any way, as the research team felt doing so could alter feasibility conclusions and potentially even modify the biochemical properties of the contents of the products themselves if opened and re‐encapsulated. Despite this design feature, the authors consider the risk of systematic unblinding to be low. Participants were not informed of the specific commercial products under study, and it would be unlikely for participants to purchase and compare the appearance, odor, and taste of the dozens of commercially available products in the CoQ10 category to the study products that they received in the trial. Although a complete‐case approach was utilized, loss to follow‐up and missing end‐of‐study laboratory results were modest, did not differ substantially across study arms, and are unlikely to meaningfully alter the results. A final limitation of the study is that the trial did not incorporate mechanistic biomarkers of the mevalonate pathway (e.g., intermediates, prenylation status of small GTPases) or GG pharmacokinetics, which limits the ability to directly test the hypothesized mechanisms underlying the lack of independent effect of GG on CoQ10 concentrations.

## 5. Conclusions

In summary, this trial provides further evidence that commercially available formulations of both ubiquinone and ubiquinol can effectively increase CoQ10 concentrations among healthy adults across the adult lifespan. This lack of statistically significant difference appeared to be robust to differences in carrier lipid content in the capsules in the products under study. While GG did not have independent effects on CoQ10 concentrations in this study and the study was not able to assess an interaction effect, there may be potential interaction in increasing CoQ10 concentrations when ubiquinol and GG are combined that could be assessed in a future factorial trial designed specifically for this purpose. Clinicians and patients may thus consider these commercially available formulations of both ubiquinone and ubiquinol at the common dose of 200 mg [32, 41, 42] to be effective options for increasing serum CoQ10 concentrations among healthy adults. The combination of this dose of ubiquinol with GG may have an interaction effect, although confirming these hypothesis‐generating findings will require a study adequately powered for this purpose, with the GG dose held constant across the GG‐alone and combination arms. Future directions of this line of research may include comparisons of other popular commercially available formulations as well as comparative evaluation of the combination of ubiquinone and GG.

## Funding

Designs for Health, Inc., Palm Coast, FL (USA), provided funding for all costs related to the research, including investigator effort, laboratory analyses, study product purchase, and participation incentives.

## Disclosure

Designs for Health had no role in the study design, data collection, statistical analysis, interpretation of results, manuscript preparation, or decision to submit the work for peer‐reviewed publication.

## Conflicts of Interest

Christopher R. D’Adamo serves on the Scientific Advisory Board for Designs for Health, Inc. The other authors declare no conflicts of interest.

## Supporting Information

Additional supporting information can be found online in the Supporting Information section.

## Supporting information


**Supporting Information** Supporting Table S1. ^∗^Mean (standard deviation). ^∗∗^ *p* values versus placebo calculated utilizing analysis of covariance (ANCOVA). ^∗∗∗^Within‐group *p* values calculated utilizing paired *t*‐tests. The supporting figure provides the labels of the products that were studied. The supporting table provides the validated questionnaire scores at baseline and at the end of the study in each of the study arms.

## Data Availability

The data that support the findings of this study are openly available in Figshare at https://doi.org/10.6084/m9.figshare.31386814.
