# Supplementary material for: A Randomized, Double‐Blind, Placebo‐Controlled Trial Comparing the Effects of Commercially Available Ubiquinone, Ubiquinol, and Geranylgeraniol Dietary Supplements on Plasma CoQ10 Concentrations
Source: J Nutr Metab. 2026 Sep 27;2026:5589963. doi: 10.1155/jnme/5589963 (PMC13617315; doi:10.1155/jnme/5589963)
Supplement: Supplementary file 1 [file JNME-2026-5589963-s001.zip › Revised Supplementary Table - RCT Comparing Ubiquinone, Ubiquinol, and Geranylgeraniol Dietary Supplements on Plasma CoQ10 Concentrations.docx]

### Supplementary Table: Questionnaire Scores at Baseline and End of Study

|  |  | ***PROMIS – Global Health – Physical Health*** | | |  |
| --- | --- | --- | --- | --- | --- |
| **Group** | **Baseline** | **End of Study** | **Change** | **p within-group*** | **p vs placebo**** |
| Ubiquinone | 52.8 (8.5) | 53.2 (9.1) | 0.4 (4.6) | 0.71 | 0.99 |
| Ubiquinol | 52.6 (7.1) | 55.8 (5.1) | 3.2 (6.3) | 0.059 | 0.16 |
| GG | 51.4 (8.4) | 53.4 (7.9) | 2.0 (4.5) | 0.071 | 0.67 |
| Ubiquinol + GG | 51.1 (5.4) | 52.5 (6.4) | 1.4 (3.6) | 0.15 | 0.94 |
| Placebo | 51.3 (8.3) | 51.5 (7.4) | 0.2 (5.0) | 0.84 | — |
|  |  | ***PROMIS – Global Health – Mental Health*** | | |  |
| **Group** | **Baseline** | **End of Study** | **Change** | **p within-group** | **p vs placebo** |
| Ubiquinone | 51.3 (8.0) | 52.7 (7.7) | 1.4 (5.8) | 0.21 | 0.99 |
| Ubiquinol | 53.7 (6.7) | 54.0 (7.9) | 0.3 (4.2) | 0.74 | 1 |
| GG | 48.5 (7.5) | 50.9 (7.2) | 2.4 (5.3) | 0.032 | 0.98 |
| Ubiquinol + GG | 50.5 (8.7) | 51.5 (7.6) | 1.0 (6.1) | 0.4 | 1 |
| Placebo | 51.4 (6.5) | 52.2 (6.5) | 0.8 (5.2) | 0.39 | — |
|  |  | ***POMS – Vigor Subscale*** | |  |  |
| **Group** | **Baseline** | **End of Study** | **Change** | **p within-group** | **p vs placebo** |
| Ubiquinone | 19.2 (6.0) | 19.2 (5.9) | 0.0 (3.7) | 0.96 | 0.44 |
| Ubiquinol | 18.5 (4.7) | 21.2 (5.2) | 2.6 (3.8) | 0.0027 | 0.99 |
| GG | 16.6 (5.6) | 18.5 (6.6) | 1.9 (4.9) | 0.06 | 0.97 |
| Ubiquinol + GG | 17.5 (4.5) | 19.8 (5.0) | 2.4 (4.8) | 0.019 | 1 |
| Placebo | 15.9 (5.1) | 18.7 (6.0) | 2.8 (4.9) | 0.0051 | — |
|  |  | ***SF-36 – Vitality Subscale*** | |  |  |
| **Group** | **Baseline** | **End of Study** | **Change** | **p within-group** | **p vs placebo** |
| Ubiquinone | 60.4 (20.2) | 63.1 (20.6) | 2.8 (12.6) | 0.26 | 0.99 |
| Ubiquinol | 60.6 (17.1) | 67.3 (16.3) | 6.7 (15.7) | 0.049 | 0.98 |
| GG | 51.1 (20.4) | 57.3 (20.5) | 6.2 (16.1) | 0.063 | 1 |
| Ubiquinol + GG | 52.7 (16.5) | 65.0 (17.8) | 12.3 (15.9) | 0.0006 | 0.68 |
| Placebo | 53.1 (18.8) | 60.0 (19.6) | 6.9 (19.1) | 0.062 | — |

* Within group p values calculated via paired t-tests. Statistical significance was defined as p<0.05.

** Comparison to placebo p values were calculated via analysis of covariance (ANCOVA). Statistical significance was defined as p<0.05.
