# Supplementary material for: A Randomized, Double‐Blind, Placebo‐Controlled Trial Comparing the Effects of Commercially Available Ubiquinone, Ubiquinol, and Geranylgeraniol Dietary Supplements on Plasma CoQ10 Concentrations
Source: J Nutr Metab. 2026 Sep 27;2026:5589963. doi: 10.1155/jnme/5589963 (PMC13617315; doi:10.1155/jnme/5589963)
Supplement: Supplementary file 1 [file JNME-2026-5589963-s001.zip › Supplementary Figure - RCT Comparing Ubiquinone, Ubiquinol, and Geranylgeraniol Dietary Supplements on Plasma CoQ10 Concentrations.docx]

# **Supplementary Figure: Labels of Study Products**

# Ubiquinone: NOW Foods - CoQ10 200 mg capsules

Ubiquinol: Jarrow Formulas - QH-Absorb 200 mg capsules

Geranylgeraniol (GG): Designs for Health - Annatto-GG 300mg

Ubiquinol + GG: Designs for Health - CoQnol 200

Placebo

| **Placebo** |  |
| --- | --- |
| Serving size | 1 softgel |
| Servings per container | 60 |
| Medium chain triglycerides | 880 mg |
| Other ingredients: Bovine gelatin, glycerine, purified water, annatto (color). | |
